# Increased kinematic changes in ascending compared with descending biplanar cut in open wedge high tibial osteotomy—a multibody simulation

**DOI:** 10.1186/s43019-024-00244-3

**Published:** 2024-11-20

**Authors:** Maximilian Jörgens, Sonja Ehreiser, Lennart Schroeder, Julius Watrinet, Wolfgang Böcker, Boris Michael Holzapfel, Klaus Radermacher, Julian Fürmetz

**Affiliations:** 1 Department of Orthopaedics and Trauma Surgery, Musculoskeletal University Center Munich (MUM), University Hospital, LMU, Munich, Germany; 2 Endogap, Joint Replacement Institute, Klinikum Garmisch-Partenkirchen, Garmisch-Partenkirchen, Germany; 3 Chair of Medical Engineering, Helmholtz Institute for Biomedical Engineering, RWTH Aachen University, Aachen, Germany; 4 Department of Trauma Surgery, BG Unfallklinik Murnau, Murnau, Germany; 5 Department of Sports Orthopaedics, Technical University of Munich, Munich, Germany

**Keywords:** Open wedge high tibial osteotomy, Biplanar cut, Multibody simulation, Biomechanics of the knee, Patellofemoral tracking

## Abstract

**Background:**

The ascending or descending extended biplanar tibial cut in open wedge high tibial osteotomy (owHTO) not only changes the lower limb anatomy in the coronal plane but also leads to different three-dimensional (3D) changes in the patellofemoral joint. This study aimed to perform a comprehensive analysis of the dynamic biomechanical changes in the knee joint using a multibody simulation model.

**Methods:**

Thirteen 3D computer models derived from lower limb computer tomography scans were used for owHTO. Osteotomies with ascending or descending biplanar cut were simulated for each wedge height from 6 to 12 mm (in 1-mm intervals). Multibody simulation was used to analyze differences in patellar shift, patellar tilt, mediolateral patellar rotation, and tibiofemoral rotation during a squat simulation from 5° to 100° knee flexion.

**Results:**

The main effects of an ascending compared with a descending extended biplanar cut in owHTO were characterized by an increase in lateralization of the patella and rotation, along with reduced tilt. Linear mixed models revealed statistically significant effects of both wedge height and cut variant on knee kinematics at 100° knee flexion, with the influence of the cut variant (ascending/descending) being higher on all analyzed kinematic parameters.

**Conclusions:**

Significant differences in the changes in patellofemoral shift, tilt, rotation, and tibiofemoral rotation were observed when performing owHTO with an ascending versus a descending biplanar cut. Apart from tibiofemoral rotation, the resulting kinematic changes were greater with an ascending cut.

## Introduction

Medial open-wedge high tibial osteotomy (owHTO) is a treatment option for varus knee osteoarthritis to avoid or at least delay total knee arthroplasty (TKA). This surgical intervention effectively redistributes the weight-bearing load from the medial to the lateral compartment of the knee joint [1, 5, 20, 22, 24, 27]. To enhance postoperative stability, surgeons routinely employ a biplanar osteotomy cut that can be extended either ascending or descending [9, 21, 25].

Notably, the ascending cut, which preserves the tibial tuberosity in the distal fragment, causes substantial alterations in the patellofemoral anatomy. It linearly increases the distance between the tibial tuberosity and the trochlea groove (TT–TG) with the extent of coronal correction [12]. Moreover, an ascending cut significantly reduces patellar height [26]. These changes associated with the ascending cut may contribute to the development of patellofemoral osteoarthritis (OA) and could potentially play a role in the occurrence of anterior knee pain following owHTO [13, 15, 16, 37].

Therefore, patients with degenerative changes in the patellofemoral joint may benefit from owHTO with a descending osteotomy cut. Studies have shown that patellar height remains unchanged and retropatellar pressure does not increase when a descending cut is performed [9, 17, 35].

Despite existing studies highlighting the effects of different biplanar cuts, a comprehensive understanding of dynamic biomechanical changes following biplanar owHTO is lacking. The objective of this study was to conduct a comprehensive analysis of the effects of an ascending versus descending extended tibial osteotomy on the dynamic biomechanics of the knee joint during a squat using a multibody simulation model. It was hypothesized that patellar shift, patellar tilt, mediolateral patellar rotation, and tibiofemoral rotation were more significantly affected by an ascending compared with a descending biplanar cut after owHTO.

## Materials and methods

### Cadaver data and 3D surface model

**Fig. 1:**
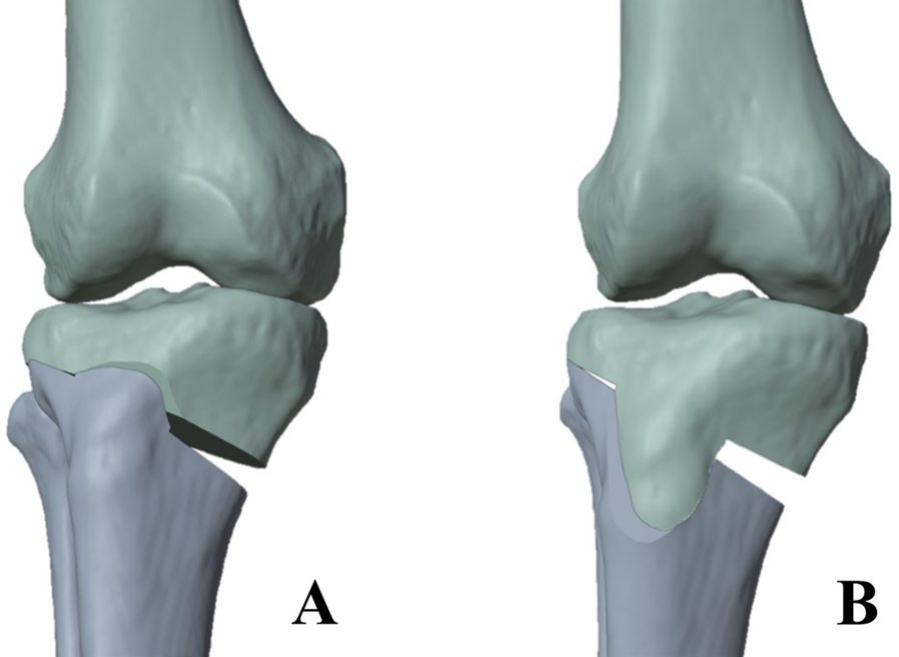
Illustration of the ascending (**A**) and descending (**B**) biplanar cut at owHTO (designed with Blender, Blender Foundation, Amsterdam, Netherlands); owHTO = medial open-wedge high tibial osteotomy

Previously acquired post mortem CT scans (standardized CT parameters, 1.25 mm slice thickness (GE HD750 CT (GE Healthcare, Chicago, IL, USA)) of 13 lower limbs were used to generate 3D surface models. Physiologically homogeneous data points were selected to test the hypothesis before pronounced deformities and more variable coronal alignments could be investigated. Exclusion criteria included advanced osteoarthritis of the hip and knee joints, joint replacement of the lower extremities, fractures or radiographic evidence of previous surgery. All patients were not older than 50 years. Ethical approval (no. 24–0099) for the use of the cadaver models was obtained according to the regulations from the institutional review board. Steps of surface model creation and osteotomy execution were based on previously published studies [7, 14]. Mimics 14.0 (Materialize, Leuven, Belgium) was used for segmentation. Models were imported into Geomagic Studio 2014 (3D Systems, Morrisville, NC, USA), and anatomic landmarks were set, then used to adjust lower limbs in the coordinate system. A strictly medial osteotomy was made parallel to the medial tibial slope. The starting point for the cut was determined after measuring the width of the tibial plateau with half of it below the tibial plateau. The owHTO was performed by using a lateral sagittal hinge axis 1.5 cm below the tibial plateau. The biplanar ascending or descending cut was added in coronal plane, 10 mm behind the tibial tuberosity, and ended accordingly cranially or distally near the tuberosity (Fig. 1).

**Fig. 2:**
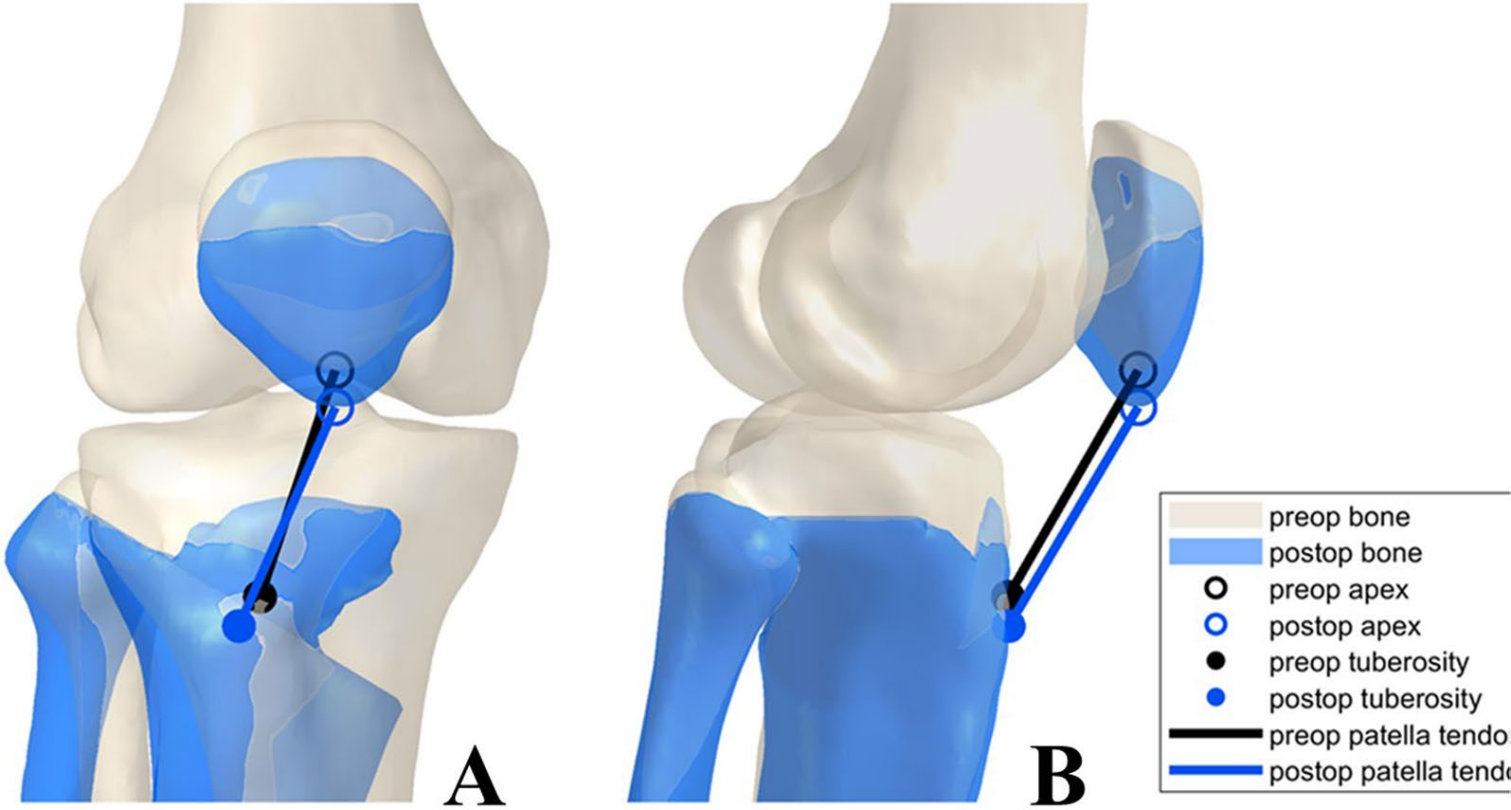
Changes in patellar tendon location and orientation. Resulting changes in patellar height with an osteotomy wedge height of 12 mm and ascending biplanar cut for the example case; A = frontal view; B = lateral view [30]

For the ascending biplanar cut models, the resulting distal shift of the patella was approximated by fixing the position in the trochlear ridge in anterior–posterior (sagittal) and medial–lateral (transversal) direction, and the longitudinal axis (proximal–distal direction) was adjusted to maintain the same patellar tendon length in the preoperative and the postoperative models. Visualizations of this method can be found both in a study by Schroeder et al. and below in Fig. 2 [30]. The owHTO was performed at increments of 1 mm from wedge height of 6 mm to 12 mm. This resulted in a total of 13 preoperative models and 182 osteotomy models (91 with an ascending and 91 with a descending extended cut).

### Multibody simulation

Using the AnyBody Modeling System™ (Anybody Technology A/S, Aalborg, Denmark), a multibody simulation of the native knee was performed [2]. The patient-specific native knee joint model was derived from an existing, validated total TKA model [2]. Osseous structures, major extensor (musculus quadriceps femoris) and flexor muscles of the knee (musculus biceps femoris and musculus semimembranosus), and ligamentous structures including collateral and cruciate ligaments were all included in the model. A separate biomechanical analysis was performed for each owHTO model, simulating knee flexion from 5° to 100°. This range of motion was selected owing to the instability of the models at full extension and in late flexion. Our simulation focuses on clinical application and is therefore limited by available input data (segmented CT imaging). Consequently, structures that require additional imaging and segmentation, such as the menisci, are neglected. We accepted this limitation, which may have led to the observed stability reduction, especially in early flexion.

**Fig. 3:**
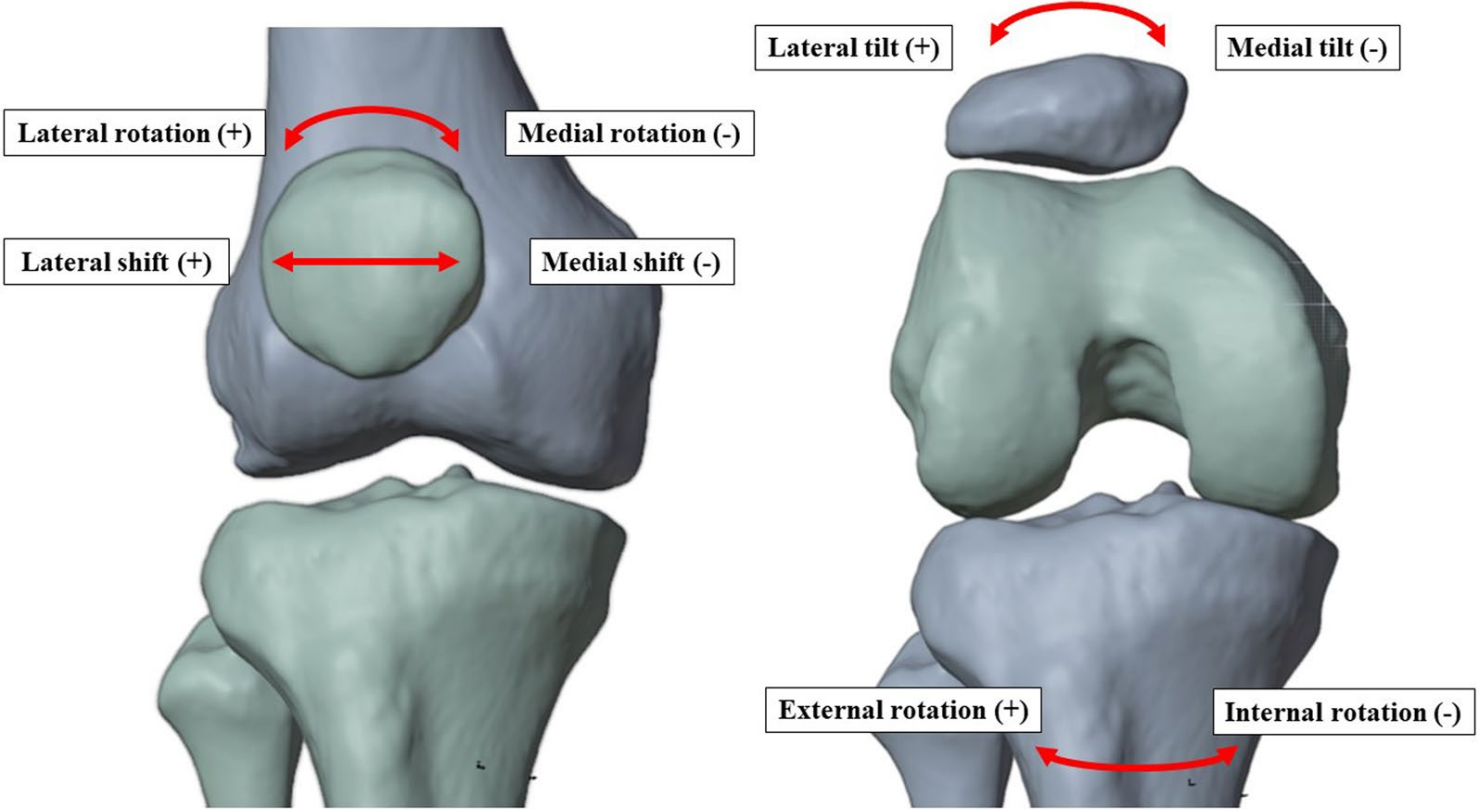
Analyzed patellofemoral and tibiofemoral parameters; (+) describes positive values (−) negative values

To define an external force in the simulation model, the patient’s body weight was estimated by the size of the femoral head according to the calculation of Ruff et al. [28]. The endpoints analyzed in this study were the comparisons of ascending and descending cuts regarding mediolateral shift, tilt, and rotation of the patella (Fig. 3). External and internal tibiofemoral rotations were also examined.

### Statistical analysis

MATLAB (The MathWorks, Inc, Natick, MA, USA) was used for statistical analysis. With each model, a squat simulation was performed and the kinematic effect at each degree of knee flexion was assessed, generating 18,720 data points in total. The root-mean-square error (RMSE) was calculated for all wedge heights. The normal distribution was investigated by visual evaluation of the simulation data at 100° knee flexion using quantile–quantile plots. *t*-Tests were performed to evaluate the differences in mean kinematic parameter values at 100° of knee flexion. Mixed models were also used to evaluate the impact of owHTO wedge height and the ascending versus the descending cut variant on knee kinematics at different flexion angles.

## Results

For a better understanding of the alignment, TT–TG, Insall–Salvati index, and hip–knee–ankle (HKA) angle, which were also part of a previous study, are shown in Table 1 [30]. Changes in the TT–TG and Insall–Salvati index only occurred with an ascending biplanar cut. HKA modification was independent of the biplanar cut.

**Table 1:** Mean values before and mean changes after owHTO according to the wedge height; standard deviation in brackets; (*) Changes in the TT–TG distance and Insall–Salvati index only in case of ascending biplanar cut (table adapted from [30])

owHTO gap	0 mm (mean)	6 mm	7 mm	8 mm	9 mm	10 mm	11 mm	12 mm
TT–TG* axial (mm)	13.6 (± 2.4)	2.1 (± 0.4)	2.4 (± 0.4)	2.8 (± 0.5)	3.2 (± 0.5)	3.6 (± 0.6)	4.1 (± 0.7)	4.5 (± 0.7)
Insall–Salvati index*	0.8 (± 0.1)	0.0 (± 0.0)	0.0 (± 0.0)	0.1 (± 0.0)	0.1 (± 0.0)	0.1 (± 0.0)	0.1 (± 0.0)	0.1 (± 0.0)
HKA (°)	178.1 (± 2.7)	5.2 (± 0.3)	6.1 (± 0.4)	7.0 (± 0.4)	7.9 (± 0.5)	8.8 (± 0.5)	9.7 (± 0.6)	10.7 (± 0.6)

The analysis found predominantly consistent trends regarding the kinematic effects of the ascending compared with the descending cut. However, the magnitude of the effects differed on an interindividual level. The patellofemoral kinematics showed greater changes in shift and tilt in owHTO with an ascending extended cut compared with a descending extended cut.

**Fig. 4:**
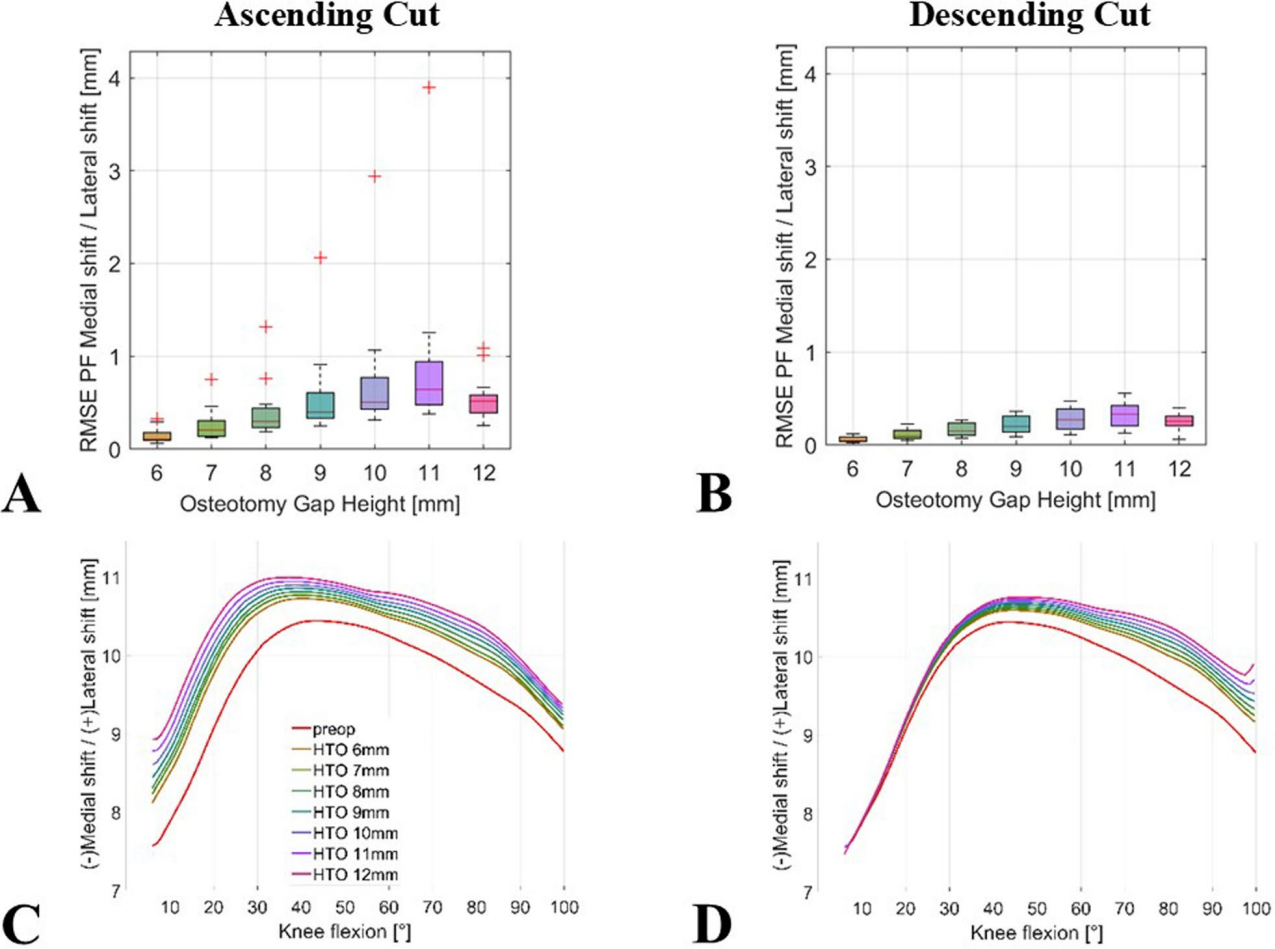
Impact of simulated owHTO on patellar shift kinematics; boxplots of mean RMSE ranges for comparison of the simulation results of the models with an owHTO of 6–12 mm wedge height and example of an individual lower limb model with native knee model (red line) and an owHTO of 6–12 mm wedge height (other lines) from 5° to 100°; (**A**) ascending and (**B**) descending biplanar owHTO cut RMSE, (**C**) ascending and (**D**) descending exemplary knee model; owHTO = medial open-wedge high tibial osteotomy, RMSE = root-mean-square error

Regarding the patellar shift in multibody simulation, an owHTO of 11 mm with an ascending cut resulted in the highest RMSE, ranging from 0.4 mm to 3.9 mm (median 0.6 mm), compared with the kinematics of the non-osteotomy model. With a descending cut, an RMSE ranging from 0.1 mm to 0.6 mm (median 0.3 mm) was observed in terms of a lateralization of the patella (Fig. 4).

**Fig. 5:**
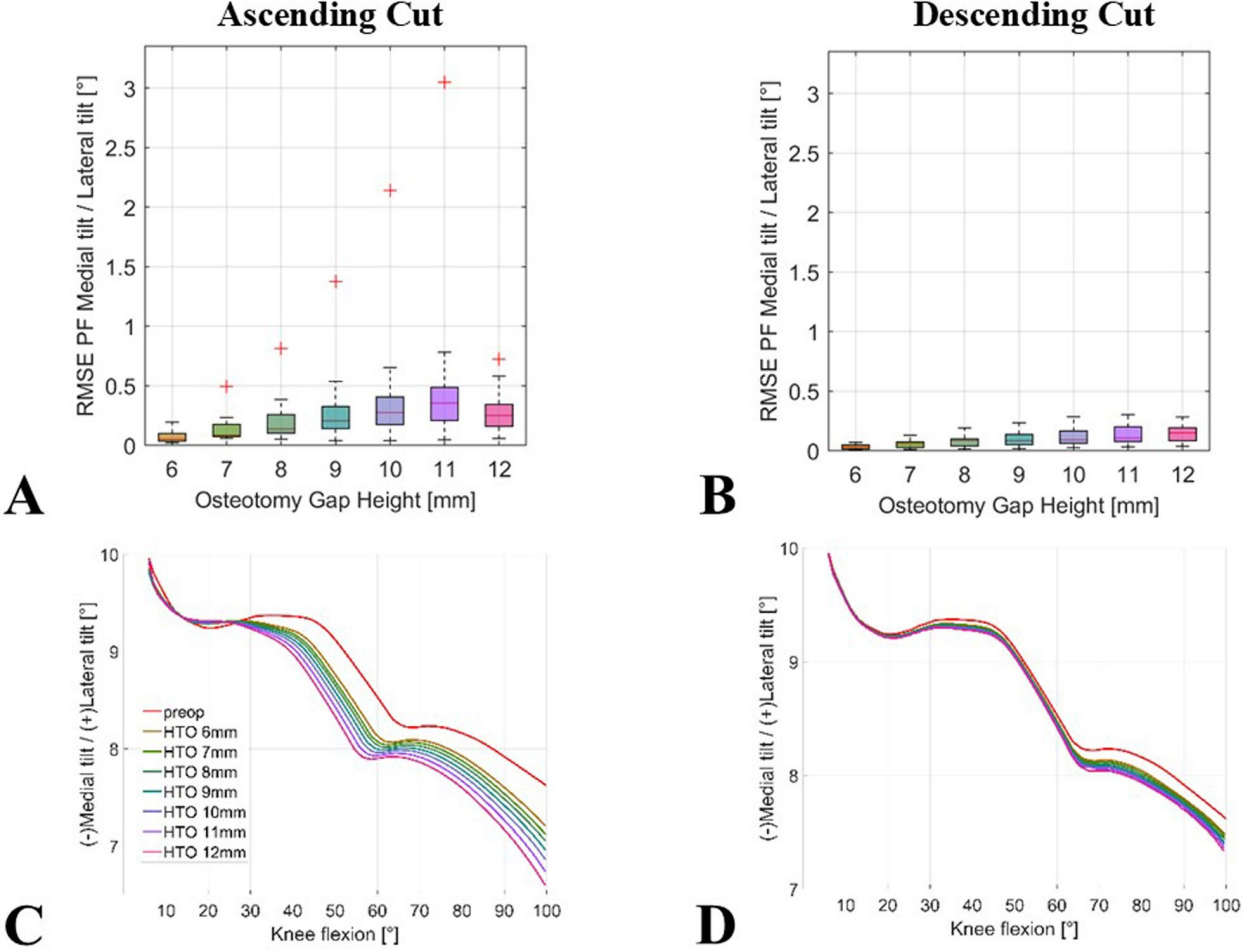
Impact of simulated owHTO on patellar tilt kinematics; boxplots of mean RMSE ranges for comparison of the simulation results of the models with an owHTO of 6–12 mm wedge height and example of an individual lower limb model with native knee model (red line) and an owHTO of 6–12 mm wedge height (other lines) from 5° to 100°; (**A**) ascending and (**B**) descending biplanar owHTO cut RMSE, ascending (**C**) and descending (**D**) exemplary knee model

Similar results were observed for patellar tilt. The RMSEs for patellar tilt were all below 0.3° (median 0.1°) for descending biplanar cut with any wedge height. In contrast, the RMSE of patellar tilt for an ascending cut ranged from 0° to 3.0° (median 0.4°) at an open wedge height of 11 mm. The exemplary curve from the simulation analysis in Fig. 5C shows that the effect on patellar tilt increases with knee flexion. With the descending extended, biplanar osteotomy, there was smaller variation to non-osteotomy models across all degrees of flexion.

**Fig. 6:**
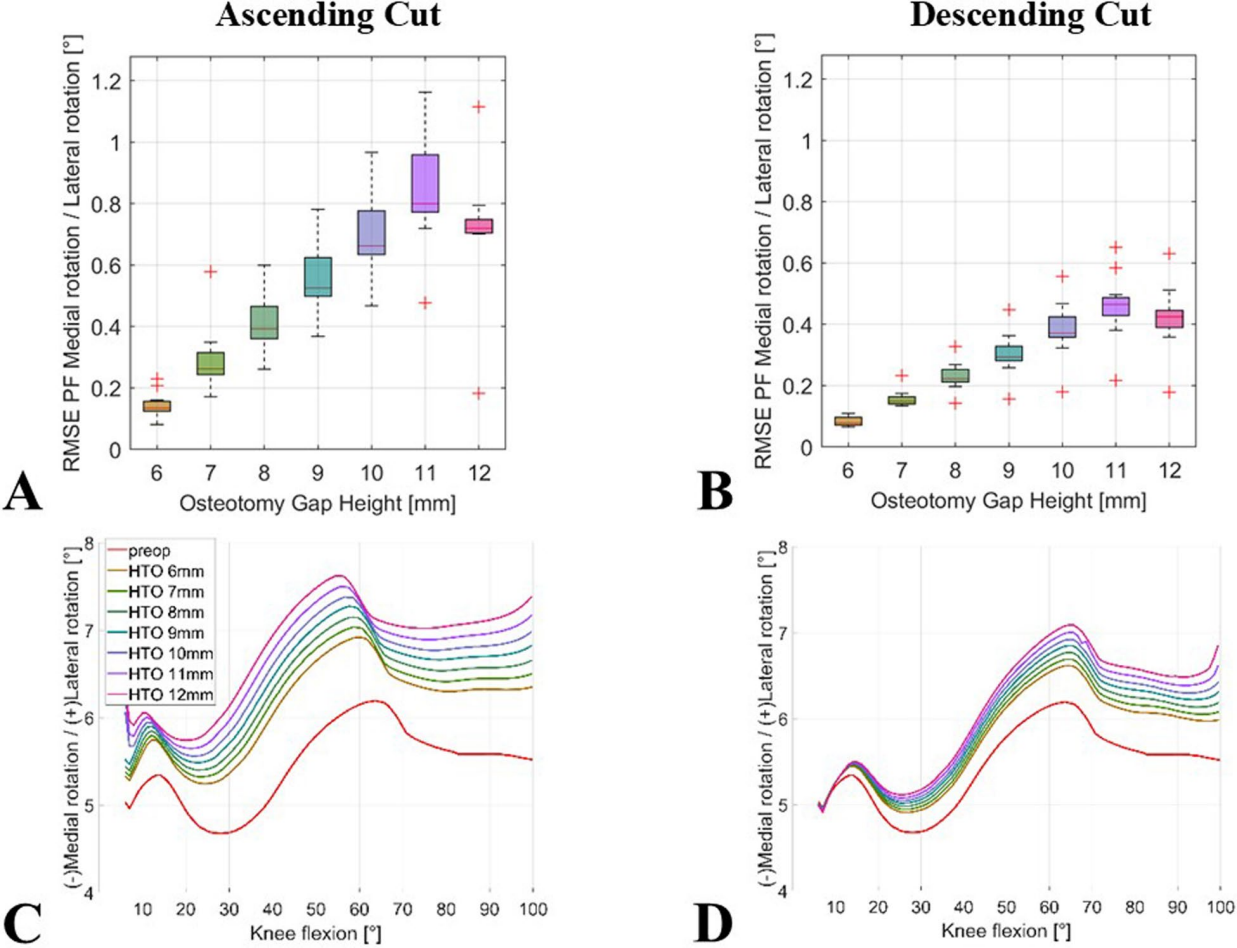
Impact of simulated owHTO on mediolateral patellar rotation; boxplots of mean RMSE ranges for comparison of the simulation results of the models with an owHTO of 6–12 mm wedge height and example of an individual lower limb model with native knee model (red line) and an owHTO of 6–12 mm wedge height (other lines) from 5° to 100°; (**A**) ascending and (**B**) descending biplanar owHTO cut RMSE, (**C**) ascending and (**D**) descending exemplary knee model; owHTO = medial open-wedge high tibial osteotomy, RMSE = root-mean-square error

Regarding patellar rotation, owHTO resulted in a higher RMSE for the ascending compared with the descending biplanar cut. The RMSE for an 11-mm wedge height ranged from 0.5° to 1.2° (median 0.8°) for the ascending cut and from 0.2° to 0.7° (median 0.5°) for the descending cut. These results are shown in Fig. 6A, B. Figure 6C, D shows a distinct deviation between ascending and descending biplanar cuts with respect to lateral patellar rotation.

**Fig. 7:**
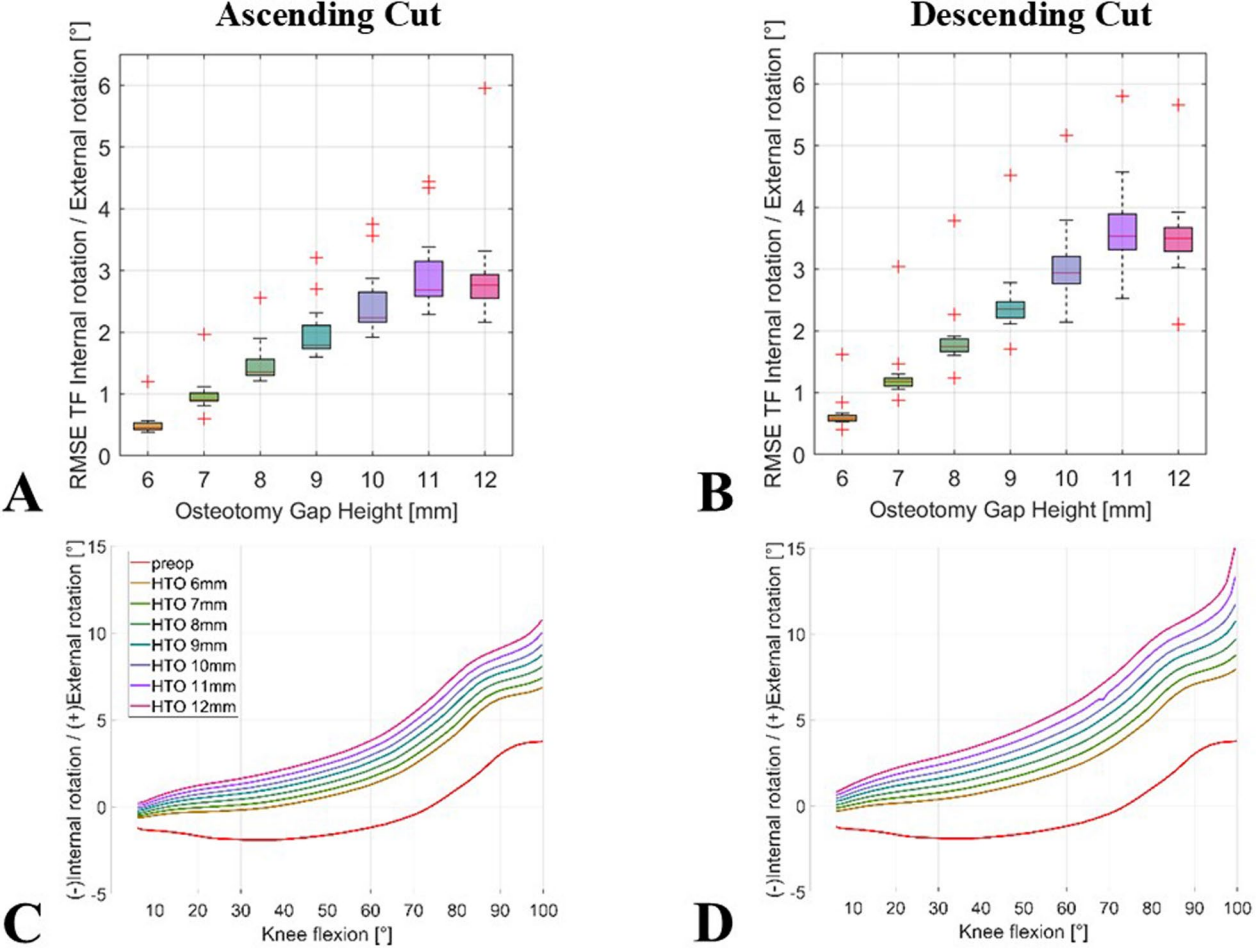
Impact of owHTO on simulated tibiofemoral rotation kinematics of an exemplary individual model; red line = before owHTO; other lines = owHTO of 6–12 mm wedge height. (**A**) ascending and (**B**) descending biplanar owHTO cut RMSE, (**C**) ascending and (**D**) descending exemplary knee model; owHTO = medial open-wedge high tibial osteotomy, RMSE = root-mean-square error

Tibiofemoral external rotation increased during flexion as a result of ascending extended osteotomy combined with increased osteotomy wedge height. However, this effect was stronger when the cut was extended descending. When performing a biplanar ascending cut with a 12 mm wedge height, the median RMSE in tibiofemoral rotation during flexion was 2.8° (range 2.2–5.9°). The analogous descending cut resulted in a median RMSE of 3.5° (range 2.1–5.7°) for tibial external rotation, when compared with preoperative values (Fig. 7A, B).

Table 2 compares the mean values for a 12 mm wedge height at 100° of knee flexion. *P*-values indicate a significant change compared with the preoperative model.

**Table 2:** Mean changes after 12 mm owHTO in 100° knee flexion depending on ascending or descending biplanar cut

Changes after 12 mm owHTO in 100° knee flexion depending on biplanar cut	Mean and SD	*P*-value
Patellofemoral shift		
Ascending	1.2 mm (± 1.8 mm)	0.0286
Descending	0.7 mm ± 0.4 mm)	< 0.001
Patellar tilt		
Ascending	−0.4° (± 1.3°)	0.2843
Descending	−0.3° (± 0.3°)	0.005
Patellar rotation		
Ascending	1.5° (± 0.5°)	< 0.001
Descending	1.0° (± 0.3°)	< 0.001
Tibiofemoral rotation		
Ascending	7.6° (± 2.9°)	< 0.001
Descending	9.3° (± 2.8°)	< 0.001

Standard deviation (SD) in brackets; corresponding *P*-values to native model

For all analyzed kinematic parameters, both the wedge height and the cut variant were found to be statistically significant predictors in linear mixed models for the mean kinematic values at 20°, 40°, 60°, and 100° of knee flexion (*P* < 0.01), with the exception of the wedge height as predictor for patellar tilt at 40° (*P* = 0.258) and 60° (*P* = 0.830). For every kinematic parameter considered in this study, the model coefficients for the cut variant were higher compared with those of the wedge height.

## Discussion

This comprehensive biomechanical analysis found that an ascending biplanar cut in owHTO yields a more lateralized, less tilted, and more laterally rotated patella under knee flexion compared with that after a descending biplanar osteotomy. Patellofemoral parameters were significantly less altered with the descending osteotomy, whereas tibiofemoral internal–external rotation was lower with the ascending osteotomy.

This study demonstrated the significant changes of patellofemoral kinematics with different biplanar owHTO techniques. These should be considered in patellofemoral OA and anterior knee pain after owHTO.

Since the publication of the owHTO technique with a descending cut by Gaasbeek et al., numerous studies have been published in recent years addressing the possible causes for anterior knee pain as well as patellofemoral OA [9, 13, 15, 16]. A stronger translation of the tibial tuberosity in the lateral direction was also found with ascending biplanar cut in owHTO. This led to an increase of the TT–TG distance [30], which is expected to result in higher patellofemoral contact forces [33]. owHTO might alter the TT–TG distance to pathologic values (≥ 20 mm), which have been associated with patellar maltracking [32]. Anatomical changes such as TT–TG alterations and kinematic tibiofemoral effects by ascending biplanar cut were also demonstrated in a previous study [30]. Apart from that, the patellar height was reduced by the more distal tibial tuberosity as well as the vectorial effective direction of the patellar tendon. Hence, the risk for patella baja was increased and may result in pathological kinematics and loading, which should be considered in owHTO [6, 8, 19, 23, 30, 31, 39]. The patellofemoral contact forces may therefore be increased postoperatively by the distal and lateral shift of the tibial tuberosity and could thereby cause higher rates of patellofemoral OA after ascending owHTO [8, 16, 35].

The present dynamic analysis found a decrease in patellar tilt after owHTO in ascending cut, which additionally showed that wedge height and flexion have an impact on lateral patellar shift, tilt, and rotation. Gaasbeck et al. also found a decrease in lateral patellar tilt with increasing ascending owHTO wedge in an in vitro study [8]. They demonstrated more lateral patellar rotation with increasing ascending owHTO wedge, which agrees with the results of this study [8].

Since there was no tuberosity translation in descending biplanar osteotomy, there were only minimal changes in the postoperative patellofemoral joint kinematics of patellar shift, tilt, and rotation. Consistent with the results of previous studies, patellofemoral kinematics was less altered and more physiological when performing the descending cut [4, 8, 18, 38]. Patella baja and possible increased retropatellar contact pressures due to kinematic changes could thus be avoided thanks to soft tissue forces and new leg alignment.

In contrast, tibiofemoral rotation could only be influenced a certain amount by an ascending or descending osteotomy. In 2014, Stief et al. described that varus malalignment was associated with increased tibiofemoral internal rotation [34]. Likewise, increased external rotation could be assumed after valgization caused by owHTO. Asseln et al. showed that a lateralized tibial tuberosity led to internal tibial rotation or reduced external rotation in flexion [3]. It was assumed that lateralization of the tuberosity by musculus quadriceps femoris led to increased traction with consecutive internal rotation of the tibia. Accordingly, a descending cut without a change in the tuberosity led to greater external tibiofemoral rotation by comparison [3].

In clinical studies, it was emphasized that patients with OA changes of the patellofemoral joint might profit from descending osteotomy biplanar cut in owHTO [10, 11, 17, 29]. Descending osteotomy might be able to minimize functional constraints in the knee and maintain the native patellofemoral joint kinematics.

### Limitations

To the authors’ knowledge, this is the first kinematic simulation study for knee flexion comparing the ascending and descending biplanar cut in owHTO. Therefore, the results of this study should be verified in biomechanical or in vivo follow-up studies. This study comes along with limitations that should be considered when analyzing the results. High interindividual differences influenced the 13 knees analyzed. Patients might profit from individual biomechanical simulations in preoperative planning, to identify an ideal owHTO variant.

The number of cases included here was limited owing to the complexity of data preparation and following simulations. With greater data gathering and simulation capacity, future investigations may be better equipped to average out interindividual differences.

Not all the models show severe varus malalignment. Since the observed trends are independent of limb alignment, it can be assumed that they also apply to varus malalignment [36]. This simulation model was modified on the basis of a previously established model for total knee arthroplasty. Validation of this owHTO model in vivo with adequate control of assumed cruciate ligament and collateral ligament elasticity is still pending. Another shortcoming of this study was that the range of knee flexion included in the analysis was limited to 5–100°. This range was set to ensure that all simulation studies could be performed and subsequently statistically analyzed, since the robustness of the model decreased at larger flexion angles.

## Conclusions

Significant differences in the changes in patellofemoral shift, tilt, rotation, and tibiofemoral rotation were observed when performing owHTO with an ascending versus a descending biplanar cut. Apart from tibiofemoral rotation, the resulting kinematic changes were greater with an ascending cut.

## Data Availability

The data that support the findings of this study are available upon reasonable request.

## Abbreviations

CT: Computed tomography
GE: General Electric
HTO: High tibial osteotomy
IL: Illinois
MA: Massachusetts
Mm: Millimeter computed tomography
NC: North Carolina
No: Number
OA: Osteoarthritis
owHTO: Open wedge high tibial osteotomy
PF: Patellofemoral
Post: Postoperative
preop: Preoperative
RMSE: Root-mean-square error
TF: Tibiofemoral
TT-TG: Trochlea groove
3D: Three-dimensional
°: Degrees

## References

[1] Agneskirchner JD, Hurschler C, Wrann CD, Lobenhoffer P (2007) The effects of valgus medial opening wedge high tibial osteotomy on articular cartilage pressure of the knee: a biomechanical study. Arthroscopy 23:852–861 17681207 10.1016/j.arthro.2007.05.018

[2] Asseln M (2019) Morphological and functional analysis of the knee joint for implant design optimization. Lehrstuhl Demberg, Germany

[3] Asseln M, Meere P, Walker P, Radermacher K (2019) Relationship of the tibial tuberosity (TT) position, tibial tuberosity-trochlear groove (TT-TG) distance, and internal and external rotation of the knee under weight-bearing conditions. CAOS 3:26–30

[4] Bito H, Takeuchi R, Kumagai K, Aratake M, Saito I, Hayashi R et al (2010) Opening wedge high tibial osteotomy affects both the lateral patellar tilt and patellar height. Knee Surg Sports Traumatol Arthrosc 18:955–960 20217394 10.1007/s00167-010-1077-5

[5] Dowd GSE, Somayaji HS, Uthukuri M (2006) High tibial osteotomy for medial compartment osteoarthritis. Knee 13(12):87–92 16515862 10.1016/j.knee.2005.08.002

[6] El Amrani MH, Levy B, Scharycki S, Asselineau A (2010) Patellar height relevance in opening-wedge high tibial osteotomy. Orthop Traumatol Surg Res 96:37–43 20170855 10.1016/j.rcot.2009.11.003

[7] Fürmetz J, Sass J, Ferreira T, Jalali J, Kovacs L, Muck F et al (2019) Three-dimensional assessment of lower limb alignment: Accuracy and reliability. Knee 26:185–193 30473372 10.1016/j.knee.2018.10.011

[8] Gaasbeek R, Welsing R, Barink M, Verdonschot N, van Kampen A (2007) The influence of open and closed high tibial osteotomy on dynamic patellar tracking: a biomechanical study. Knee Surg Sports Traumatol Arthrosc 15:978–984 17483931 10.1007/s00167-007-0305-0

[9] Gaasbeek RD, Sonneveld H, van Heerwaarden RJ, Jacobs WC, Wymenga AB (2004) Distal tuberosity osteotomy in open wedge high tibial osteotomy can prevent patella infera: a new technique. Knee 11:457–461 15581764 10.1016/j.knee.2004.02.002

[10] Gooi SG, Chan CXY, Tan MKL, Lim AKS, Satkunanantham K, Hui JHP (2017) Patella height changes post high tibial osteotomy. Indian J Orthop 51:545–551 28966378 10.4103/ortho.IJOrtho_214_17 PMC5609376

[11] Han C, Li X, Tian X, Zhao J, Zhou L, Tan Y et al (2020) The effect of distal tibial tuberosity high tibial osteotomy on postoperative patellar height and patellofemoral joint degeneration. J Orthop Surg Res 15:466 33036644 10.1186/s13018-020-01996-w PMC7547468

[12] Hodel S, Zindel C, Jud L, Vlachopoulos L, Furnstahl P, Fucentese SF (2023) Influence of medial open wedge high tibial osteotomy on tibial tuberosity-trochlear groove distance. Knee Surg Sports Traumatol Arthrosc 31:1500–1506 33891162 10.1007/s00167-021-06574-z

[13] Hunter DJ, Zhang YQ, Niu JB, Felson DT, Kwoh K, Newman A, Nevitt M (2007) Patella malalignment, pain and patellofemoral progression: the Health ABC Study. Osteoarthritis cartilage 15(10):1120–1127 17502158 10.1016/j.joca.2007.03.020 PMC2042530

[14] Jorgens M, Keppler AM, Degen N, Bachmeier AT, Bergstraesser M, Sass J et al (2022) Reliability of 3D planning and simulations of medial open wedge high tibial osteotomies. J Orthop Surg (Hong Kong) 30:10225536221101700 35694778 10.1177/10225536221101699

[15] Kalichman L, Zhu Y, Zhang Y, Niu J, Gale D, Felson DT, Hunter D (2007) The association between patella alignment and knee pain and function: an MRI study in persons with symptomatic knee osteoarthritis. Osteoarthr Cartil 15(11):1235–1240 10.1016/j.joca.2007.04.014 17570690

[16] Kataoka K, Watanabe S, Nagai K, Kay J, Matsushita T, Kuroda R (2021) Patellofemoral osteoarthritis progresses after medial open-wedge high tibial osteotomy a systematic review Arthroscopy. J Arthrosc Relat Surg. 37(10):3177–3186 10.1016/j.arthro.2021.04.015 33895305

[17] Krause M, Drenck TC, Korthaus A, Preiss A, Frosch KH, Akoto R (2018) Patella height is not altered by descending medial open-wedge high tibial osteotomy (HTO) compared to ascending HTO. Knee Surg Sports Traumatol Arthrosc 26:1859–1866 28417183 10.1007/s00167-017-4548-0

[18] Lee YS, Lee SB, Oh WS, Kwon YE, Lee BK (2016) Changes in patellofemoral alignment do not cause clinical impact after open-wedge high tibial osteotomy. Knee Surg Sports Traumatol Arthrosc 24:129–133 25288336 10.1007/s00167-014-3349-y

[19] Lenhart RL, Brandon SC, Smith CR, Novacheck TF, Schwartz MH, Thelen DG (2017) Influence of patellar position on the knee extensor mechanism in normal and crouched walking. J Biomech 51:1–7 27939752 10.1016/j.jbiomech.2016.11.052 PMC5204307

[20] Lind M, McClelland J, Wittwer JE, Whitehead TS, Feller JA, Webster KE (2013) Gait analysis of walking before and after medial opening wedge high tibial osteotomy. Knee Surg Sports Traumatol Arthrosc 21(21):74–81 21484389 10.1007/s00167-011-1496-y

[21] Lobenhoffer P, Agneskirchner JD (2003) Improvements in surgical technique of valgus high tibial osteotomy. Knee Surg Sports Traumatol Arthrosc 11:132–138 12774149 10.1007/s00167-002-0334-7

[22] Niemeyer P, Stohr A, Kohne M, Hochrein A (2017) Medial opening wedge high tibial osteotomy. Oper Orthop Traumatol 29:294–305 28642979 10.1007/s00064-017-0509-5

[23] Otsuki S, Murakami T, Okamoto Y, Nakagawa K, Okuno N, Wakama H et al (2018) Risk of patella baja after opening-wedge high tibial osteotomy. J Orthop Surg (Hong Kong) 26:2309499018802484 30295136 10.1177/2309499018802484

[24] Ozel O, Yucel B, Mutlu S, Orman O, Mutlu H (2017) Changes in posterior tibial slope angle in patients undergoing open-wedge high tibial osteotomy for varus gonarthrosis. Knee Surg Sports Traumatol Arthrosc 25:314–318 25763850 10.1007/s00167-015-3571-2

[25] Pape D, Lorbach O, Schmitz C, Busch LC, Van Giffen N, Seil R et al (2010) Effect of a biplanar osteotomy on primary stability following high tibial osteotomy: a biomechanical cadaver study. Knee Surg Sports Traumatol Arthrosc 18:204–211 19809806 10.1007/s00167-009-0929-3

[26] Ren YM, Tian MQ, Duan YH, Sun YB, Yang T, Hou WY (2022) Distal tibial tubercle osteotomy can lessen change in patellar height post medial opening wedge high tibial osteotomy? A systematic review and meta-analysis. J Orthop Surg Res 17:341 35794572 10.1186/s13018-022-03231-0 PMC9258196

[27] Rossi R, Bonasia DE, Amendola A (2011) The role of high tibial osteotomy in the varus knee. J Am Acad Orthop Surg 19:590–599 21980024 10.5435/00124635-201110000-00003

[28] Ruff CB, Holt BM, Niskanen M, Sladek V, Berner M, Garofalo E et al (2012) Stature and body mass estimation from skeletal remains in the European Holocene. Am J Phys Anthropol 148:601–617 22639191 10.1002/ajpa.22087

[29] Sahanand KS, Pandian P, Chellamuthu G, Rajan DV (2023) Effect of ascending and descending medial open wedge high tibial osteotomy on patella height and functional outcomes-a retrospective study. Eur J Orthop Surg Traumatol. 10.1007/s00590-023-03693-w 37632547 10.1007/s00590-023-03693-w

[30] Schroeder L, Grothues S, Brunner J, Radermacher K, Holzapfel BM, Jorgens M et al (2024) Open wedge high tibial osteotomy alters patellofemoral joint kinematics: a multibody simulation study. J Orthop Res. 10.1002/jor.25945 39080850 10.1002/jor.25945

[31] Smoger LM, Fitzpatrick CK, Clary CW, Cyr AJ, Maletsky LP, Rullkoetter PJ et al (2015) Statistical modeling to characterize relationships between knee anatomy and kinematics. J Orthop Res 33:1620–1630 25991502 10.1002/jor.22948 PMC4591110

[32] Steensen RN, Bentley JC, Trinh TQ, Backes JR, Wiltfong RE (2015) The prevalence and combined prevalences of anatomic factors associated with recurrent patellar dislocation: a magnetic resonance imaging study. Am J Sports Med 43:921–927 25587185 10.1177/0363546514563904

[33] Stephen JM, Lumpaopong P, Dodds AL, Williams A, Amis AA (2015) The effect of tibial tuberosity medialization and lateralization on patellofemoral joint kinematics, contact mechanics, and stability. Am J Sports Med 43:186–194 25367019 10.1177/0363546514554553

[34] Stief F, Bohm H, Dussa CU, Multerer C, Schwirtz A, Imhoff AB et al (2014) Effect of lower limb malalignment in the frontal plane on transverse plane mechanics during gait in young individuals with varus knee alignment. Knee 21:688–693 24725590 10.1016/j.knee.2014.03.004

[35] Stoffel K, Willers C, Korshid O, Kuster M (2007) Patellofemoral contact pressure following high tibial osteotomy: a cadaveric study. Knee Surg Sports Traumatol Arthrosc 15:1094–1100 17342550 10.1007/s00167-007-0297-9

[36] Watrinet J, Joergens M, Blum P, Ehmann Y, Augat P, Stuby F et al (2024) Tibial tuberosity-trochlear groove distance is significantly decreased by medial closing wedge distal femoral osteotomy. Knee Surg Sports Traumatol Arthrosc 32:287–294 38270286 10.1002/ksa.12053

[37] Wright JM, Heavrin B, Begg M, Sakyrd G, Sterett W (2001) Observations on patellar height following opening wedge proximal tibial osteotomy. Am J Knee Surg 14:163–173 11491427

[38] Yang JH, Lee SH, Nathawat KS, Jeon SH, Oh KJ (2013) The effect of biplane medial opening wedge high tibial osteotomy on patellofemoral joint indices. Knee 20:128–132 23127422 10.1016/j.knee.2012.09.019

[39] Yang JS, Fulkerson JP, Obopilwe E, Voss A, Divenere J, Mazzocca AD et al (2017) Patellofemoral contact pressures after patellar distalization: a biomechanical study. Arthroscopy 33:2038–2044 28844344 10.1016/j.arthro.2017.06.043

